# Assessing the impact of stigma reduction interventions in Iran: A qualitative study from the perspective of mental health stakeholders

**DOI:** 10.3389/fpubh.2022.1027002

**Published:** 2022-12-08

**Authors:** Ahmad Hajebi, Seyed Sepehr Hashemian, Moussa Abolhassani, Amirali Hajebi, Kamyab Alizadeh, Amir Mohsen Rahnejat, Mojgan Khademi, Arsia Taghva

**Affiliations:** ^1^Research Center for Addiction and Risky Behaviors (ReCARB), Psychosocial Health Research Institute, Iran University of Medical Sciences, Tehran, Iran; ^2^Department of Psychiatry, Faculty of Medicine, Iran University of Medical Sciences, Tehran, Iran; ^3^Ph.D in Psychology, Department of Psychology, Allameh Tabataba'i University, Tehran, Iran; ^4^International Federation of Inventors' Associations-IFIA, Geneva, Switzerland; ^5^Cognitive and Behavioral Research Center, Aja University of Medical Sciences, Tehran, Iran; ^6^Non-communicable Diseases Research Center, Endocrinology and Metabolism Research Institute, Tehran University of Medical Sciences, Tehran, Iran; ^7^School of Medicine, Aja University of Medical Sciences, Tehran, Iran; ^8^Department of Clinical Psychology, Faculty of Medicine, Aja University of Medical Sciences, Tehran, Iran; ^9^Department of Psychiatry, School of Medicine, Shahid Beheshti University of Medical Sciences, Tehran, Iran; ^10^Cognitive and Behavioral Research Center, School of Medicine, Aja University of Medical Sciences, Tehran, Iran

**Keywords:** stigmatization, challenges, mental health, Iran, qualitative study, help seeking behavior

## Abstract

**Introduction:**

The fear and embarrassment associated with stigmas discourage patients from help seeking behavior, which may explain why even the patients' loved ones advise them to discontinue treatment to avoid being labeled. In addition, stigmas can lead to personal and family issues, causing patients to disregard their illness. As such, their disease may develop into a chronic condition. This being said, the present study aims to investigate the challenges, solutions, and successes associated with stigmatization in Iran from the perspective of mental health stakeholders.

**Method:**

A qualitative study was conducted in the summer of 2022. Purposive sampling was utilized to recruit participants. The primary data collection method involved a focus group interview that lasted 110 min. The project manager monitored the interviews, and all research team members attended the meetings, took notes, and made the necessary preparations. After explaining the study's purpose and ensuring the data's confidentiality, the interviewer led a focus group discussion. The interviews were recorded with the participants' consent. A focus group was used to conduct interviews with 13 individuals until data saturation was reached.

**Findings:**

Ten psychologists, psychiatrists, and managers responsible for mental health, two patients, and one patient's family member participated in the current study as eligible participants. Repeated readings led to the emergence of three main classes under the headings of *challenges, solutions*, and *successes* of stigma management in Iran, each containing subclasses.

**Conclusion:**

The majority of the proposed solutions in this study centered on raising awareness and training diverse individuals and groups to lessen stigmas. The most crucial de-stigmatization measure is to offer training that will cause the current stereotypes to change. This must be taken by patients and their families as well as therapists, leaders, policymakers, the general public, and the media. Ideally, younger members of the target groups should be considered for these pieces of training, which must be based on research and derived from cultural and localized needs.

## Introduction

The human body and mind are intimately linked and interdependent. Therefore, the disease affecting one also affects the other. The effects and symptoms of physical diseases are typically perceptible with the eyes and understandable through the five senses. However, mental and nervous diseases typically affect a person's emotions, feelings, and behavior such that in the early stages of the disease, there is a vague feeling and state that the patient cannot understand easily. Initially, a patient with a mental problem feels incompatibility with social expectations and ideals because of the disease's symptoms and complications. As a result, the patient feels ashamed of and disappointed with him/herself. To alleviate this disparity, many patients try to conceal their condition. Nonetheless, they ultimately experience a loss of their civil, social, and human rights. Patients with neurological and mental disorders typically face two major challenges. First, they must manage the symptoms of their illness, which can vary depending on the type of the disease (such as anxiety, delirium, hallucinations, etc.). These symptoms can affect the affected individual's employment, independence, and life satisfaction. Second, the erroneous perception of society can result in (social) stigmas among these individuals (1, 2).

Throughout history, psychiatric diseases have been consistently associated with incorrect beliefs and deviant social reactions to the extent that people with psychiatric diseases were believed to be possessed by the devil. Indeed, the majority of psychiatric disorders provoke a negative impression in the general public, resulting in stigmas and social problems (3).

The word stigma originally referred to a mark applied to Greek slaves to differentiate them from free individuals. As such, stigma was a highly discrediting characteristic. In Persian, however, the word stigma denoted a symbol written on commercial documents. Additionally, Scambler has classified stigmas into two categories: emotional (self-stigmatization) and contractual (discrimination) (4).

A stigma influences the development of mental disorders to such an extent that researchers recognize it as the most significant risk factor and barrier to improving mental health. No aspect of the mental health system is stigma-free; patients, families, and healthcare providers, as well as the diagnosis and treatment of mental illnesses, have always been subject to criticism, protest, and discrimination (2–5).

A stigma is defined as a negative stereotypical view combined with bigoted ideas and discrimination, which results in job, livelihood, and communication losses for the patient and those around the patient due to the unfair perception it creates. The stigmas associated with mental illnesses are the greatest barrier to care provision. Stigmas affect not only patients but also their families and therapists. Even facilities that offer treatment, psychotherapeutic medications, and mental health professionals are not immune to stigmas. Stigmas cause society and decision-makers to dismiss mental illness. Hence, they tend to lack a coherent and purposeful plan to solve the problems of mentally ill patients and are reluctant to seek out and explore resources to assist people with mental disorders. In addition, this stigma results in discrimination when providing services to individuals with both physical and mental disorders (6, 7).

Numerous studies have demonstrated the prevalence of negative beliefs about people with mental health conditions. Stigmas distance individuals from social standing and human dignity. These negative views are prevalent in many nations. In Nigeria, for instance, the general populace holds numerous false beliefs about people with mental health conditions, such as being dangerous, unpredictable, repulsive, and useless to society (8–10).

Also, a stigma exists in every aspect of life, including the workplace and the classroom. It is assumed that patients with severe mental illness (such as psychosis-bipolar disorders and severe depression) are stigmatized to a greater degree. Alonso et al. demonstrated that people with minor psychiatric disorders such as anxiety might also be stigmatized (11, 12).

Unfortunately, patients with mental diseases endure stigma from a variety of sources, including society, surrounding people, and even mental healthcare providers. These negative beliefs about mental illnesses are pervasive throughout society, and they appear to be deeply rooted in culture and reinforced and perpetuated by popular culture and media. Prejudices and biases related to mental health and people with mental illnesses are frequently associated with unrealistic expectations. These unrealistic expectations create conditions for the patient whereby they internalize the stigmas, leading to further stigmatization in society. In this case, people with mental illness are stigmatized not only by society, family, colleagues, and mental healthcare professionals but also by themselves (13–15).

Stigmas also transform a normal individual into one who is unstable and broken. Stigmas cause a person with a mental illness to feel ashamed and to perform less well in society. In addition, the low self-esteem of these individuals leads to difficulties in finding a job and a home, accessing the justice system, and utilizing health support services, ultimately leading to greater social isolation. Various studies demonstrate that stigmas bring about a decline in patients' self-esteem and performance. Furthermore, stigmas negatively affect the treatment process, including help seeking behavior, accepting psychotherapy, and so on. A study conducted in Norway revealed that only 13% of depressed individuals and 25% of those with anxiety disorders seek treatment. According to Corrigan, stigmas are recognized as the most significant barrier to mental patient referral (16–18).

Multiple studies have examined the negative effects of stigmas on patients and their families. In the study conducted by Phillips et al., moderate and severe stigma-related effects were observed in 60% of patients and 26% of families. In Iran, Shahveisi et al. found that 32% of families with schizophrenic members and 12% of families with depressed members concealed the disease from others. In Sadeghi's study, 16% of families with a schizophrenic member, 37% of those with a depressed member, and 26% of families with members suffering from bipolar disorder concealed the family member's illness. In this study, the stigma intensity increased when the disease duration was longer than 2 years and there was more than one hospitalization instance. In the study by Shahveisi et al., 12% of families reported workplace discrimination. In another study, nearly half of adults expressed embarrassment at the prospect of seeing a psychiatrist (13, 19, 20).

The fear and embarrassment of stigma discourage patients from help seeking behavior, which is why even the patient's loved ones may advise them to discontinue treatment so as to avoid being labeled. In addition, stigmas may lead to personal and family issues, causing patients to disregard their illness and experience more chronic conditions over time. To avoid bearing the stigma of mental illness, a patient may seek care from non-psychiatric specialists. Due to stigmas, patients often request that psychiatrists not use their insurance cards for psychiatric drugs. Patients with mental illness may also feel ashamed of their families' financial support for treatment due to the associated stigma (2, 21, 22).

Despite the high number of mental disorders and people in need of counseling in the country, very few people seek counseling and treatment from psychiatrists; this may suggest that Iran bears more stigmas than other countries. A further reason for non-referral could be patients' absence of social support. Additionally, we have observed that patients suffer greatly from the stigma of the disease due to the negative evaluation of patients by some research scholars and social leaders. Lastly, the slow stigmatization process and lagging behind international efforts in terms of stigmatization have exacerbated the situation. As such, the present study aims to investigate stigmatization in Iran. Challenges, solutions, and successes are assessed from the perspective of mental health stakeholders.

## Methods

This research is a qualitative investigation conducted during the summer of 2022. A purposive sampling method was utilized. The primary data collection method involved a focus group interview that lasted 110 min. The project manager monitored the interviews, and all research team members attended the meetings, took notes, and made the necessary preparations. After explaining the study's purpose and ensuring the data's confidentiality, the interviewer led the focus group discussion. The interviews were recorded with the participants' prior consent. A focus group was used to conduct interviews with 13 individuals until data saturation was reached.

The field notes that were recorded immediately after the interviews were reviewed, and the interviews were concurrently transcribed verbatim. Regarding the number of interviews, the primary criterion for the researcher was the use of key informants, the data itself, emerging classes and theory, and theoretical saturation. Field notes, unstructured observations, and written narratives constituted additional data collection instruments. The researchers concurrently collected, coded, and analyzed the data. Corbin and Strauss's coding paradigm (2008) was drawn upon for data analysis. This methodology involved three stages of analysis: open, central, and selective coding. In addition, the constant comparison method was utilized throughout all analysis phases, and differences and similarities between the primary codes were identified. Similar codes were conceptualized and assigned to the same category. The codes were reviewed continuously in a back-and-forth manner and revised as necessary. Comparing data, posing questions, writing the primary storyline, drawing diagrams, and reviewing reminders were among the techniques employed during the coding process.

Before conducting the research, the questions were analyzed to ensure they were congruent with the research objectives. In addition, a consensus was reached on the research context, data collection method, and research ethics. Using the participant confirmation method in the data analysis was one way to ensure the credibility of the present study. In addition, the extracted codes and themes were reviewed and approved by the researcher and two additional university professors who were familiar with the qualitative research approach and were authorities in the field of stigmas.

In this context, the transferability of the extracted codes was discussed and confirmed by the researchers above. The anonymity principle was the most crucial concern for the researchers. Per the ethical codes of the Declaration of Helsinki (2013) and the American Sociological Association (2001), this study respected the participants' consent to participate, participant anonymity, and the use of alternative names in reports and articles. This study was approved by the Ethics Committee of Iran University of Medical Sciences, Tehran, Iran (code: IR.IUMS.REC.1398.824).

## Results

In accordance with the data saturation criterion, 13 individuals participated in the current study, including 10 psychologists, psychiatrists, and managers in charge of mental health, two patients, and one patient family member. Repetitive readings led to the extraction of three main classes under the headings of *challenges, solutions*, and *successes* of dealing with stigmas in Iran. Each of these main classes has sub-classes as displayed in Table 1.

**Table 1:** Classes, sub-classes, and basic concepts extracted from the data.

**Basic concepts**	**Sub-classes**	**Classes**
Attitude of the general public	Beliefs, attitudes, and insufficient awareness	Challenges
General ignorance of society		
Education, children, and mental health		
Lack of teaching resources for students		
Negative view of psychiatrists and mental health therapists	Mental health workers and other professionals	
Stigmas among doctors (general and specialist) and paramedics		
Problems of patients and families with the disease		
Non-accepting patients in general hospitals		
The government, the Ministry of Health, and the non-cooperation of organizations	Structures and policymakers	
Lack of a coherent program		
Problems in (social-therapeutic) structures		
Stigmatization of psychiatry		
NGOs		
Insufficient financial resources of families and economic problems	Insufficient financial resources	
Non-cooperation of benefactors		
Lack of insurance support		
Budget		
Culture	Cultural barriers	
Media		
Literature and works of art		
Concealment and failure to provide statistics		
Statistics-based needs analysis	Research-based and evidence-based measures	Solutions
Modeling successful projects		
The starting point of destigmatization		
Training and changing the attitude of healthcare providers	Emphasis on education and attitude change	
Public education and raising awareness		
Beginning of education from childhood		
Using the potential of clerics and scholars		
Using the potential of the media	Cultivation	
Training of media personnel		
Use of books and educational materials		
Popular culture		
The role of social networks		
Holding festivals		
The role of prominent people		
Launching a campaign and appointing a support ambassador		
Highlighting and introducing well-managed patients		
Using common literature and creating new literature		
Using the word “nerve” instead of “psychiatrist” in panels		
Budget	Support services and coverage	
The necessity of proper pricing for psychiatric and psychological services		
Insurances		
Formation of a committee and a secretariat	Integrated reform of structures -and policies	
Demarcation of disciplines and preventing the intrusion of non-specialists		
Integration of psychiatric departments in general hospitals		
Determine the guardian of mental health		
Emphasis on having a written and comprehensive plan		
Attention to the social rights of patients		
Reforming the public perspective in providing services		
The need to support patients and families		
The necessity of inter-organizational support and coordination and avoiding rework		
Using available resources to destigmatize		
NGOs and independent organizations	The role of different organizations and institutions	
The role of the Ministry of Health		
The role of the Ministry of Science		
The role of the Ministry of Guidance		
The role of the Ministry of Education		
The role of the municipality		
The role of Saman advertizing		
The need to use the potential of different organizations		
Office of mental health	Policies	Successes
Mental health management		
The link between the private and public sectors	Treatment	
Increasing the number of psychologists and psychiatrists		
Placing the board of psychiatrists next to other specialties		

### Challenges

#### Beliefs, attitudes, and insufficient awareness

##### Attitude of the general public

Inappropriate attitudes among social strata have contributed to stigmas, leading to the general public's erroneous perception of hospitalized people with mental health conditions. One of the causes of stigmas is, for instance, society's failure to recognize schizophrenia—a condition they have heard about but are unaware of its nature. This lack of recognition strengthens negative attitudes, which is a barrier to changing people's perspectives on mental illness. The absence of a favorable scientific attitude among the general population and society's incorrect reaction to the presentations of mental and atypical diseases, including schizophrenia, create a sense of fear and unpredictability among patients, resulting in increased stigmas toward mental illnesses and the subsequent increased pain and suffering of the patient due to insulting labels.

##### General ignorance of society

The public's ignorance and lack of knowledge regarding mental illnesses are regarded as significant destigmatization barriers. Indeed, in Iran, a significant number of people turn to non-specialists such as fortune-tellers, palm-readers, and magicians rather than mental health specialists to solve mental health problems.

##### Education, children, and mental health

One of the bedrocks of stigmatization in society is the lack of attention to mental health in children's education and the incorrect teaching of mental illnesses to children at the community level.

##### Lack of teaching resources for students

Despite the sensitive role of students in society and the fact that the future of society is infused with them, no curricula or resources are focusing on mental diseases and disorders. Even the Ministry of Science opposes implementing a two-credit mental health course for students at all levels.

#### Mental health workers and other professionals

One of the major impediments to stigma reduction in Iran has been the sometimes ineffective role of mental health workers and even professionals working in the mental health field.

##### Negative view of psychiatrists and mental health therapists

The absence of shared literature among mental health therapists, particularly psychologists and psychiatrists, and the entry of non-specialists into the mental health realm are among the obstacles to destigmatizing mental illnesses. The lower income of mental health specialists relative to other health specialties was cited as one of the existing problems, which has led to boredom and even fostered a negative perception among psychiatrists and, more specifically, psychologists.

##### Stigmas among doctors (general and specialist) and paramedics

Inadequate training of physicians regarding mental disorders and negative attitudes among physicians and paramedics have contributed to stigmas associated with mental illnesses. The inappropriate treatment of people with mental health conditions by medical staff and the negative attitudes of doctors and support institutions toward people with mental health conditions are additional obstacles to destigmatization. In addition, the lack of adequate training for paramedics regarding mental health and the disregard of some physicians for mental disorders contribute to the perpetuation of this stigma.

##### Problems of patients and families with the disease

Long-term familial involvement and treatment courses of these patients are among the contributors to society's stigmatization of patients with mental conditions, thereby perpetuating the stigmas.

##### Non-acceptance of patients in general hospitals

General and public hospitals do not admit psychiatric patients, nor do the emergency rooms.

#### Structures and policymakers

##### The Ministry of Health, the government, and non-cooperation of organization

Participants cited inadequate cooperation between scientific centers as an impediment to decreased stigmatization. The lack of coordination between organizations pertaining to mental health, the limited involvement of the Ministry of Health in mental health, and the absence of unified action among organizations and planners contribute to the stigmatization of mental illness in society. The Ministry of Health has omitted mental health issues from its list of priorities due to a lack of support from policymakers and planners.

##### Lack of a coherent program

The absence of a national strategy for stigma reduction and the lack of coordinated anti-stigma efforts at the national level have prevented the development of effective destigmatization initiatives. One of the factors contributing to the persistence of stigmas against people with mental health conditions is the absence of a comprehensive plan and effective actions to combat them.

##### Problems in (social-therapeutic) structures

Due to the complexity of urban structures and the prevalence of mental illness, problems are escalating and structural flaws are becoming more apparent. For instance, the absence of halfway houses as a viable treatment method is more apparent now than in the past. On the other hand, the disconnection between medical centers and society leads to the spread of stigmas. Moreover, due to existing limitations and gaps, the inadequate response of medical centers results in the patient's social isolation.

##### Stigmatization of psychiatry

The lag in introducing mental health to the general public, the non-socialization of psychiatry, the stigmatization of terms such as psychologist and psychiatrist, the restriction of mental health to clinics, and the vagueness of the institution in charge of mental health in society are among the major problems with mental health promotion in Iran leading to the spread of stigmas.

##### NGOs

Another challenge associated with mental health in Iran is the small number of non-governmental organizations (NGOs), the authorities' disregard for them, and the lack of operational power of the NGOs.

#### Insufficient financial resources

##### Insufficient financial resources of families and economic problems

According to some participants, financial resources play an important role in providing services and reducing the stigma associated with mental illnesses in the contemporary world. Insufficient financial resources, the high cost of psychiatric treatments, and economic issues that prevent people with mental health conditions from receiving treatment are, from a family member's perspective, the most significant problems. Accordingly, there might be non-referrals, whereby the disease deteriorates and takes on a worse presentation. Meanwhile, outpatient and inpatient treatment costs are only partially covered in Iran.

##### Non-cooperation of benefactors

Donors and charitable foundations are among the major constituents of the Iranian healthcare system. However, a lack of donor cooperation in the field of mental health is one of the obstacles to destigmatization. Donors have made fewer strides or are unwilling to collaborate in this field.

##### Lack of insurance support

Patients and their families are concerned about insurance companies covering the costs of long-term treatment for mental illnesses. Given the large number of patients, insurance companies resist covering medical expenses. In addition to medication and hospitalization costs, insurance companies have partially covered psychotherapy services in the past year, which is a positive development. Insurance companies are not mandated to fully cover mental health problems, giving rise to further stigmas associated with mental illnesses.

#### Budget

The appropriate budget line for resolving or mitigating a problem is a trustees' indication of its significance. Participants in the study hypothesized that the reason for concealing the exact number of people with mental health conditions is the constant desire to reduce the mental health budget; they viewed this as a grave injustice against this defenseless portion of society. Among other obstacles, it is possible to mention the inadequate knowledge of actual needs, the inadequacy of the current budget, the economic disorganization and officials' negligence, and the high number of people with mental health conditions, which prevents adequate budgeting for patients.

#### Cultural barriers

##### Culture

Culture, which functions as a double-edged sword, is a significant factor in reducing or creating stigma in any given society. Most participants viewed culture as a platform wherein a lack of strength tends to increase stigmas. Culture has taken a long time to reach us. Equally reasonable, its transformation will also require considerable time. In addition, perfectionism is one of the defining features of our culture. In this culture that does not tolerate flaws, mental illness is regarded as a major and intolerable shortcoming.

#### Media

The media is another influential cultural contributor to mental illness-associated stigmas. The media's insufficient knowledge of psychiatric illnesses is one of the factors that contributes to stigmas in society and causes patients to endure double suffering. The non-scientific nature of some topics raised in the media and the non-specialized terms employed in the media portray people with mental health conditions as frightening and unpredictable. Unfortunately, this misinterpretation of psychiatric illnesses by the media is prevalent in many nations. On the other hand, there have been few successful media campaigns to destigmatize individuals with certain mental illnesses. In Iran, the national media's inability to destigmatize is evidenced by the absence of appropriate scripts and the propagation of false beliefs. In addition, in Iran, programmers rarely use scientific consultants during film production, which has led to bizarre and unrealistic depictions of people with mental health conditions.

##### Literature and works of art

Recent years have seen a significant increase in concerns regarding the words of artists and authors concerning mental disorders, particularly the non-scientific use of terms such as schizophrenia in published literature. In our literature, a mental illness diminishes a person's worth, whereas a physical illness does not, and this discrimination between different patients contributes to a negative perception of mental illnesses. In our literary culture, terms such as mental, crazy, and stupid are typical examples of derogatory terminology.

##### Concealment and failure to provide statistics

Some authorities' emphasis on the secrecy and concealment of mental illnesses is one of the most significant obstacles to comprehending the true scope of mental problems in society. Society's desire to conceal mental illnesses and the absence of an honest and realistic assessment of the current situation are prime examples of a lack of understanding of the actual needs in this field. The Iranian culture of perfectionism compels families to conceal a loved one's disease and casts doubt on the patient's way of life.

### Solutions

#### Research-oriented and evidence-based measures

The foundation of any macro executive action is research. Clearly, an effective strategy to reduce stigma can be achieved by relying on previous research and evidence-based planning.

##### Statistics-based needs analysis

The communication of needs based on statistics facilitates precise planning. The available data and studies should be evaluated, and a needs analysis should be conducted. One also needs to pay close attention to the cultural components of society so that an effective plan can be devised to address the enormous stigma problem.

##### Modeling successful projects

Using successful models from around the world and neighboring nations, the cooperation with the UNESCO chair in Iran, and the destigmatization of the AIDS program and addiction as examples, it is possible to implement successful programs here as well.

#### Emphasis on education and attitude change

Modifying people's attitudes can be cited as an important strategy for reducing stigma. Some participants in the study underlined the importance of belief and attitude shifts prior to providing funding to destigmatize mental illnesses. Among these measures is correcting false beliefs within the medical community and even among mental healthcare providers.

##### Training and changing the attitude of healthcare providers

*General physicians, specialists, paramedics, and other therapists*. Evidently, physicians play a key role in the initial assessment of referring patients and in introducing the field of psychiatry. Importantly, too, they are also capable of altering people's attitudes. Appropriate alteration of attitudes appears to be a factor that must be incorporated into studies and curricula. Destigmatizing mental illnesses, therefore, requires working with medical students, correcting the views of general practitioners and specialists, and justifying non-psychologists and non-psychiatrists. It is crucial for medical students not to be influenced by their pre-existing mentality but rather to examine and treat mental illnesses using scientific evidence. In addition to general practitioners, one should be mindful of the beliefs formed in the minds of other specialists, and their attitudes should be assessed during their academic training.

*Psychiatrists and psychologists*. In addition to their well-known function, mental health therapists play an essential role in reducing stigma. Improving the quality of psychiatric services is one measure psychologists and psychiatrists take to lessen the stigmas associated with mental illnesses. Comprehensive and team-based treatment is one of these methods. Holding classes and workshops for the general public and other healthcare workers can help reduce stigmas and their appearance in the media. Effective stigmatization-related measures include providing optimal services for mental health workers, providing effective and immediate treatment to prevent the worsening of patients' conditions for a better presence in society, and providing comprehensive and correct treatment.

##### Public education and awareness

Participants in the study highlighted public education and awareness, specialized education during one's studies, and offering a two-credit mental health course to all university students as effective factors in destigmatizing mental illnesses. In addition, the education of kindergarten teachers, patients' families, peers, and the general public; the continuous preparation of brochures and booklets; and the emphasis on the biological etiology of diseases raise the level of literacy and awareness of individuals and special populations and reduce stigmas.

##### Beginning of education from childhood

According to the participants, it is possible to shape children's attitudes from an early age by beginning work in preschool and to incorporate life skills lessons into preschool programs. Beginning schooling at a young age reduces the stigmas associated with people suffering from mental health conditions.

##### Using the potential of clerics and scholars

An undeniable fact is the influence of clerics and scholars on the general populace, which may benefit destigmatization. Seminaries, the Islamic Propaganda Organization, the Office of Friday Prayer Imams, and congregations are appropriate venues for destigmatization. The use of religious podiums for Friday prayers can be effective for this purpose. Religious elders' religious teachings and traditions demonstrate their leadership in lessoning stigmas against people with mental health conditions.

#### Cultivation

The culture of a society, which derives from its customs, traditions, and beliefs, is a fundamental and enduring issue. Basic planning that leads to new cultures will be valuable for reducing the stigma associated with mental illnesses.

##### Using the potential of the media

Multiple surveys indicate that the media plays an irreplaceable and undeniable role in promoting or mitigating stigmas. It is possible to utilize the extensive capacity of the media to educate the audience accurately and alter their false beliefs. For instance, by creating educational videos, stigmas can be effectively alleviated. On the other hand, attitudes can be improved by organizing a festival of psycho-friendly films. Conversely, we can improve literature and reduce the stigmas associated with mental illnesses by utilizing artists and media specialists. It is preferable, however, to take this measure under the supervision of experts so that stigmatization does not occur in society due to these programs.

##### Training of media personnel

Considering the important role of media in reducing stigmas, it may prove highly beneficial to train planners and program makers. However, the implementation of these programs is associated with problems. The radio and television trustees must be determined to implement it in practice.

##### Use of books and education materials

It is expected that steps will be taken to reduce stigmas by preparing a variety of written works, as books and educational materials have a profound and influential effect on the audience. It should be noted, however, that the preparation of these items is not limited to a specific time or day of the year and that professional writers must be requested to create them; mental healthcare professionals are not permitted to pick up a pen themselves.

##### Popular culture

Attention to common and general beliefs and cultures is vital to mitigating stigmatization. Consequently, reforming public culture and establishing cultural committees to establish new terms are essential. It is recommended to move forward with those who have worked in this field. Similarly crucial is the participation of various public and private sectors in cultural work.

##### Social networks

Given the ever-increasing growth of social networks and their unique impact on the general public, it is expected that a significant portion of the impact on the general public can be achieved through social networks. These networks are widespread and equipped with various facilities, making them an ideal medium for audience education.

##### Holding festivals

In order to highlight a certain issue, numerous festivals with the potential for great influence are held around the globe. Thereby, the audience's attention is drawn to these programs, and appropriate stigma-reduction strategies can be implemented. While festivals have proved effective globally, there is room in our country to consider preparations in this area. Participants in the study emphasized the importance of holding various festivals for a variety of general or specialized audiences. These festivities may be cultural, artistic, or athletic. Moreover, it is possible to exhibit works created by patients or works about patients at art festivals. As such, psychological issues become commonplace to the public.

##### The role of prominent people and celebrities

Today, many charitable endeavors benefit from the assistance and company of prominent and famous individuals. The support of these individuals will increase social awareness about mental illnesses and effectively change public attitudes. Many participants emphasized the use of this method in destigmatization programs. This way, famous athletes, national heroes, artists, and politicians can contribute effectively.

##### Launching a campaign and appointing a support ambassador

Efficient advertizing campaigns are based on research and analysis of consumer needs. The establishment of think tanks and unified organizations will greatly assist these campaigns. In the current study, numerous stakeholders expressed willingness to provide their facilities for stigma reduction campaigns. A further suggestion was to appoint a popular mental health ambassador interested in participating in such programs.

##### Highlighting and introducing well-managed patients

Reducing stigma is accomplished by highlighting and utilizing the experiences of successful and well-managed patients. By sharing their experiences, these individuals can serve as a model for other patients, their families, and the public. This will diminish the distorted and disappointing image that has already formed in the minds of the audience.

##### Common literature and creation of new literature

Today, one single body of literature and common terminology is utilized within every discipline. Unfortunately, in Iran, due to the negative nature of some words and their valorization, there is a need to form common and new literature among specialists so that the patient is not addressed with words that carry a negative connotation. Further, words such as soul, which is related to religion, should be avoided, and there should be consistency in word usage.

#### Supportive services and covers

##### Budget

The need for financial support and the allocation of adequate funds, as well as the need for equipment and logistical support in the fight against stigma, necessitates that the authorities allocate sufficient funds to fight stigma.

##### Necessity of proper pricing for psychiatric and psychological services

To avoid discipline discrimination and provide appropriate services to patients, the value of psychological services should be recognized on par with the value of other medical services. Even special attention should be directed to psychological services.

##### Insurances

It was suggested that the insurance umbrella be made more specific and thorough in order to lessen stigmas and offer patients better services. Chronic people with mental health conditions were specifically suggested to be included in the special patient's category.

#### Integrated reform of structures and policies to improve the performance of mental health trustees

##### Formation of a committee and a secretariat

Establishing a committee to collect data in accordance with the World Psychiatric Association's plans and emphasizing the formation of a headquarters or a strategic council are among the top stigmatization solutions. This organization has a unique organizational structure that is required for a coordinating team to establish specialized and operational committees that can support patients with mental problems. Participants emphasized the need for a secretariat with a well-defined schedule to avoid duplication and complete a large national undertaking.

##### Demarcation of disciplines and preventing the intrusion of non-specialists

It is necessary to clarify and define interdisciplinary boundaries and prevent unqualified and non-specialized individuals from interfering. As long as roles and positions are not clearly defined, the course of events may be derailed.

##### Integration of psychiatric departments in general hospitals

Important destigmatization measures include expanding psychiatric units in general hospitals, consolidating psychiatric departments in general hospitals, and establishing psychosomatic (psycho-physical) departments in general hospitals. Iranians are credited with the first integration of psychiatric departments in general hospitals, as evidenced by historical records. Currently, according to the legal resolution allocating 10% of beds in general hospitals to people with mental health conditions, a substantial amount of progress has been made in terms of stigma reduction.

##### Determining the guardian of mental health

According to the law, the Ministry of Health, Care, and Medical Education is the official guardian and planner for mental health. Occasionally, however, it is necessary to make this role more distinct and prominent so as to separate responsibilities and define the scope of work.

##### Emphasis on having a written and comprehensive plan

With a planned and codified program at the macro level, stigmatization can begin in both fundamental and specialized areas. This work necessitates the presence of a comprehensive and accurate plan, as well as the notification of the relevant institutions. With the right macro-level policy and unity of action, it is possible to develop stigmatization programs that avoid duplication of efforts and parallel work in different organizations.

##### Attention to the social rights of patients

The provision of more sophisticated facilities for mentally ill patients will normalize their demands for equal rights in the eyes of the general public, allowing them to be regarded as normal citizens. Since patients require a normal life, it is necessary to modify the structure and relations in order to guarantee their social rights.

##### The need to support patients and families

Social acceptance of patients reduces their likelihood of rejection. Support from organizations improves their companionship and acceptance. The establishment of daily service centers staffed by specialists such as psychiatrists, psychologists, occupational therapists, social workers, and psychiatric nurses is crucial for accepting patients and their families. Thus, the programs must be comprehensive enough to encompass all patients from various groups and strata. In the interim, it is essential to respect and safeguard patients' privacy.

##### Necessity of inter-organizational support and coordination and avoiding rework

Personnel and operational support, inter-organizational cooperation, observance of material and moral rights, and elucidation of responsibilities are crucial. To reduce stigmas, it would be preferable if the Ministry of Health, Care, and Medical Education took advantage of these facilities while recognizing the personnel-operational potentials in various organizations. Clearly, in these inter-organizational programs, more results could be achieved with a smaller budget by avoiding duplicate work.

#### The role of different organizations and institutions

In addition to the Ministry of Health, Care, and Medical Education and the Welfare Organization, this study discovered that numerous and dispersed centers in Iran could assist individuals with mental problems, including the municipality, the Islamic Propaganda Organization, and the Scientific Association of Health Education, among others.

Healthy individuals are the first line of defense in the fight against stigmas. This is necessary for people with mental health conditions due to the presence of non-governmental organizations and support centers for physical insurance. In this context, the *Association of Poets, Artists, and Writers*, the *Centre for Intellectual Development*, the *Book Council*, and NGOs and the organizational formation of NGOs can be effective.

##### The role of the Ministry of Health

As the official guardian of mental health, the Ministry of Health should have strong executive arms; as a result, the mental health office should be bolstered, and the mental health structure should be improved and given a greater role.

##### The role of Welfare Organization and the Ministries of Guidance, Science, and Education

Together with the Ministry of Health, these ministries and organizations can play a significant role in destigmatization by creating jobs, collaborating in education provision, and publishing books and articles.

##### The role of the municipality

Utilizing the potential of the municipality and its close ties and neighborhood-based connections with the people, as well as health houses and centers, is extremely beneficial in destigmatizing mental illnesses.

##### The role of the Islamic Propaganda Organization

This organization can hold a unique position in reducing stigmatization, given its wide scope of activities, the presence of clerics in the majority of cities and remote villages, and the availability of suitable facilities and infrastructures for widespread advertizing.

##### The need to use the potential of other organizations

Participants emphasized the importance of leveraging the potential of military systems, kindergartens, mosques, seminaries, telecommunications, parliamentarians, and Friday prayer imams.

Successes**:**

We have also made progress in policy and treatment due to the recent practices of the Ministry of Health and the shift in the attitude of managers and government officials over the past decade.

#### Policy

- Formation of mental health offices and their independent management.
- Mental health used to be primarily concerned with diagnozing disorders; however, it now also focuses on disease prevention.
- Mental health services cover residents in cities and villages alike.

#### Treatment

- More activity of psychologists and psychiatrists.
- Presence of a panel of psychiatrists along with other specialists.
- Links between private and public sector.

## Discussion

This study revealed that stigmas had been the subject of relatively few studies in Iran. Indeed, the literature portrays only a portion of the actual situation. For instance, the study conducted by Ghanean et al. focuses solely on Tehran and not the entire country (23).

As demonstrated by Fiorillo et al.'s study of 27 countries and 108 European organizations, stigma is one of the top treatment priorities for psychiatrists, mental health therapists, medical officials, and non-governmental mental health organizations. In the present study, all participants agreed that addressing stigmas and the resulting discrimination is an essential component of national mental health programs. Since no serious and effective planning has been performed in this regard—destigmatization—there is a strong need to address it. Participants believed that stigmas constituted a major factor in the lack of referrals and discrimination against people with mental health conditions. From the perspective of the participants in this study, who represented important and influential governmental and non-governmental sectors, stigmas are so important that they are willing to cooperate practically, seriously, and actively in national infrastructure programs to reduce stigmas and combat the associated discrimination (24, 25).

No aspect of our psychiatry is stigma-free, according to the study participants. Similar to research elsewhere, this study found that this problem affects not only neurotic or psychotic patients but also their families. Moreover, therapists in this field, such as psychiatrists and other mental illness therapists, are also susceptible to these biases. Moreover, we have observed negative attitudes regarding other aspects of mental health, such as psychiatric diagnoses and classifications, which have always been the subject of discussion and debate (20, 26, 27).

Psychiatric treatments are stigmatized, particularly medication, ECT, electroshock, and hospitalization. In the current study, as in the study reported by Ozman et al., the complications and challenges of the treatment are highlighted, but the treatment's positive effect is not mentioned (28, 29).

Similar to McSwain, we have observed discrimination and stigmas in mental disorder policies, service provision, insurance coverage, and budget allocation. Participants in this project viewed the current study as an example of the steps they expected to be taken in the country's comprehensive fight against stigmas. This study comprehensively examines the intricate and intertwined structure of stigmas in an effort to provide practical solutions to combat them (7, 30).

As in a study by Volpio et al., the participants in the current study, including psychiatrists, officials, families, and patients, reiterated that patients initially seek help from non-psychiatrist therapists for mental health problems. While consultation with non-psychiatrist therapists is less stigmatic, delayed treatment with a psychiatrist can make the disease's progression more chronic and difficult. Indeed, Kolshaw et al. noticed that non-psychiatry specialists do not include medical details on their consultation sheets for psychiatrists (31, 32).

Non-specialists abusing the patient's condition under the guise of prayer writers, fortune tellers, and exorcists complicate the treatment process, according to the participants in the study. The widespread presence of this group of unlicensed and unofficially practicing healers, particularly on the outskirts of cities, is one of the primary causes of delayed patient referral. Apparently, this interest in traditional healers parallels findings from two studies: those conducted by Akighir et al. and Alem et al. (33, 34).

The participants cited the perfectionist and idealistic culture in Iranian society, which hardly tolerates shortcomings, contributing to the intensification of stigmas. This perspective finds more challenging conditions and a more pessimistic outlook concerning mental disorders as typically long-term health problems. This perspective has possibly led to peculiar concealment of information, statistics about the current situation, and even secrecy at the level of families and patients such that patients may even refuse to employ the available treatment facilities. It appears that a portion of the desire to conceal the disease among patients and their families, as well as by some professionals, lies in the cultural characteristics of the society. Moreover, in a culture that views every flaw, no matter how minor, as major and sometimes as an unforgivable sin, it is evident that mental disorders, whether they be a brief anxiety disorder or a severe problem such as schizophrenia, are both looked down upon. Indeed, prior quantitative studies conducted in Iran have discovered that approximately one-third of families tended to conceal the disease from others.

One of the limitations of this research is the interview with the decision makers in this area, so it is recommended to examine the factors affecting stigma from the perspective of patients and their families as well as experts present in the treatment system.

## Conclusion

The majority of the solutions presented in this study centered on raising awareness and training various individuals and groups to reduce stigmas. The most important action, both on the level of therapists, the general public, leaders, policymakers, and the media, as well as among patients and their families, is to provide training that will result in the modification of previously held stereotypes. Ideally, younger members of the target groups should be considered for these pieces of training, which must be based on research and derived from cultural and localized needs.

In addition to official authorities such as the Ministry of Health and the Welfare Organization, this study found that organizations and centers in Iran can indirectly contribute to stigma reduction programs. At first glance, it may seem preferable to consolidate these centers under the supervision of a trustee. However, depending on the policies in place, the presence and company of influential groups can be utilized to develop psychological programs. On the other hand, it was discovered that some locations in Iran are active or potentially willing to cooperate in providing services to psychiatric patients. Therefore, the formation of a committee or office to coordinate various programs and treatments (likely within the Ministry of Health as the primary steward of mental health policy) can be advantageous.

One of the positive effects of destigmatization or the reduction of stigmas is improved quality of life and, hence, higher credibility and elevated dignity of the patients and their families before the general public. As such, these measures will demonstrate that these patients have rights and abilities comparable to those of healthy members of society and that they can express their desires without fear of being judged by others. Under such circumstances, they will live a relatively normal life, legal institutions will value their abilities, the general public will have a more favorable opinion of them, and stigmas will be less visible. Secondly, there are social benefits. Assisting vulnerable patients to demonstrate their abilities and promoting them as successful individuals at the societal level help their abilities to emerge, and society may benefit from their productive presence. Chronic diseases may be prevented, and social problems such as unemployment and delinquency may be reduced to a great extent if this occurs. Thirdly, we may notice economic growth and cost reduction. The successful implementation of stigma reduction programs lowers the financial burden placed on families of mentally ill patients, the economic losses incurred by organizations due to the depreciation of the workforce, and the cost of providing care for people with mental illness. Social and therapeutic measures become more balanced, and the treatment cost cycle improves.

Undoubtedly, an important issue such as the stigma of the disease, which in addition to the individual, the family and the society are also involved in this issue, needs to be investigated and conducted more research to understand its dimensions, characteristics and consequences in order to consider the necessary measures for prevention. and increase the treatment. It is hoped that the findings of this research will be the basis for conducting more research in this field so that a deeper understanding of the concepts of the findings can be carefully determined and used.

## Data availability statement

The original contributions presented in the study are included in the article/supplementary material, further inquiries can be directed to the corresponding authors.

## Author contributions

AhH and AT have contributed in conducting the research. MK, SH, and AmH has performed the data analysis. AhH, AT, SH, AR, AmH, KA, MA, and MK have contributed to inscribing the main body of the article. All authors contributed to the article and approved the submitted version.
